# Commonly disrupted pathways in brain and kidney in a pig model of systemic endotoxemia

**DOI:** 10.1186/s12974-023-03002-6

**Published:** 2024-01-04

**Authors:** Kimberly C. Olney, Camila de Ávila, Kennedi T. Todd, Lauren E. Tallant, J. Hudson Barnett, Katelin A. Gibson, Piyush Hota, Adithya Shyamala Pandiane, Pinar Cay Durgun, Michael Serhan, Ran Wang, Mary Laura Lind, Erica Forzani, Naomi M. Gades, Leslie F. Thomas, John D. Fryer

**Affiliations:** 1 Department of Neuroscience, Mayo Clinic, 13400 East Shea Boulevard, Scottsdale, AZ USA; 2 Mayo Clinic Graduate School of Biomedical Sciences, Scottsdale, AZ USA; 3 MD/PhD Training Program, Mayo Clinic, Scottsdale, AZ USA; 4 Division of Nephrology & Hypertension, Mayo Clinic, 13400 East Shea Boulevard, Scottsdale, AZ USA; 5 School of Engineering of Matter, Transport & Energy, Arizona State University, Tempe, AZ USA; 6 Department of Comparative Medicine, Mayo Clinic, Scottsdale, AZ USA

**Keywords:** Systemic inflammation, Sepsis, Septic, Shock, Lipopolysaccharide, LPS, Infection, Organ dysfunction, Acute kidney injury, Pig model, Porcine model, Bulk RNA-seq, Brain impairment, Transcriptome, Immune system, Immune regulation

## Abstract

Sepsis is a life-threatening state that arises due to a hyperactive inflammatory response stimulated by infection and rarely other insults (e.g., non-infections tissue injury). Although changes in several proinflammatory cytokines and signals are documented in humans and small animal models, far less is known about responses within affected tissues of large animal models. We sought to understand the changes that occur during the initial stages of inflammation by administering intravenous lipopolysaccharide (LPS) to Yorkshire pigs and assessing transcriptomic alterations in the brain, kidney, and whole blood. Robust transcriptional alterations were found in the brain, with upregulated responses enriched in inflammatory pathways and downregulated responses enriched in tight junction and blood vessel functions. Comparison of the inflammatory response in the pig brain to a similar mouse model demonstrated some overlapping changes but also numerous differences, including oppositely dysregulated genes between species. Substantial changes also occurred in the kidneys following LPS with several enriched upregulated pathways (cytokines, lipids, unfolded protein response, etc.) and downregulated gene sets (tube morphogenesis, glomerulus development, GTPase signal transduction, etc.). We also found significant dysregulation of genes in whole blood that fell into several gene ontology categories (cytokines, cell cycle, neutrophil degranulation, etc.). We observed a strong correlation between the brain and kidney responses, with significantly shared upregulated pathways (cytokine signaling, cell death, VEGFA pathways) and downregulated pathways (vasculature and RAC1 GTPases). In summary, we have identified a core set of shared genes and pathways in a pig model of systemic inflammation.

**Supplementary Information:**

The online version contains supplementary material available at 10.1186/s12974-023-03002-6.

## Highlights

- Robust inflammatory response in pig brain following LPS, with significant differences from a similar mouse model.
- Shared genes and pathways in the brain and kidneys following LPS in a pig model.
- Characterization of gene-level and isoform-level transcriptional alterations in blood, brain, and kidney post-LPS challenge in a pig model.

**Supplementary Information:**

The online version contains supplementary material available at 10.1186/s12974-023-03002-6.

## Introduction

Sepsis is a life-threatening condition, stimulated most commonly by bacterial and other infections, characterized by the host's dysregulated inflammatory response which involves the evolution of complex, time-dependent processes including both proinflammatory cytokines and a compensatory anti-inflammatory response [1–5]. Sepsis may be viewed as one potential stage along a continuum of immune adaptations with the broader range of responses including systemic inflammatory response syndrome (SIRS), sepsis, severe sepsis, and septic shock [6]. Sepsis is characterized by impaired cellular oxygen utilization despite adequate oxygen delivery with cytopathic hypoxia recognized as a mechanism of resulting organ dysfunction [7, 8].

Systemic inflammation commonly results in acute kidney injury (AKI). Systemic inflammation-associated AKI plays a substantial role in the morbidity and mortality of sepsis [9, 10]. The comorbidity of sepsis is high among all cases of AKI; up to 50% of AKI instances are associated with sepsis, and up to 60% of patients with sepsis also have AKI [11–13]. While the pathophysiologic mechanism remains incompletely understood, recent studies have suggested that the deleterious inflammatory cascade of sepsis may contribute to AKI [14], and septic AKI is increasingly being recognized as a heterogenous syndrome, consisting of sub phenotypes [12].

In addition to the kidneys, multiple tissues, including those of the central nervous system (CNS), may be damaged by the complex milieu arising from sepsis conditions [4, 15, 16]. While activation of innate immunity is necessary to combat pathogens, over-activation is damaging [17–19]. Several proinflammatory cytokines (e.g., IL-1 $\beta$, IL-6, TNF-$\alpha$) are observed during the progression from systemic inflammatory response syndrome to septic shock [6, 20]. This sepsis-associated “cytokine storm” often induces significant CNS dysfunction, producing altered mentation acutely. Sepsis survivors often develop chronic cognitive and behavioral impairments, indicating that sepsis may produce significant and lasting neurological consequences [3, 21–25].

Multiple studies have investigated endotoxemia response in large mammals [26–28]. Terenina et al. conducted a longitudinal survey of LPS response in pigs, assessing blood transcriptomic, hormonal, and metabolic reactions, and identified an overall immune response in swine with cortisol levels reaching peak 4 h post-LPS injection [27]. Bush et al. compared the gene expression response of sheep bone marrow-derived macrophages to LPS at various time points and found a conserved transcription factor network shared with humans [28]. Gene expression profiles of sheep bone marrow-derived macrophages at 0 and 7 h post-LPS treatment were compared across different large mammals, revealing a shared macrophage functional transcriptome [28]. Bush et al. (2020) further showed that large mammals differed from rodents in the inducible expression of genes related to arginine metabolism and nitric oxide production [28]. Although these studies have enhanced our understanding of endotoxemia response in large mammals, much of our understanding of the molecular and cellular changes in affected tissues during the septic response has come from mouse models [29, 30]. Relative to rodents, pigs demonstrate a more greatly shared physiology to that of humans (especially including renal physiology) [31], more similar immune system to that of humans [32], and large gyrencephalic brain anatomy which more closely approximates that found in humans [33, 34]. Thus, we developed a porcine model to advance our understanding of how systemic inflammation impacts molecular and cellular changes in specific tissues, including kidney, brain, and blood.

We administered intravenous (IV) lipopolysaccharide (LPS), a component of bacterial cell walls commonly used to model the inflammatory aspects of sepsis [17, 35], to female *Sus scrofa* Yorkshire pigs and profiled the prefrontal cortex of the brain, kidney, and whole blood by bulk RNAseq. We applied a gene-level and isoform-level analysis to identify transcriptional alterations post-LPS challenge. Compared to saline controls, pigs that received LPS exhibited numerous known and novel genes and pathways impacted in these early stages of the hyperinflammatory response, with striking differences between the brain response and published data from a similar mouse model. We identified a core set of changes in both brain tissue and kidneys that implies a shared organ response that was not observed in whole blood. Additionally, many genes without differences in gene expression showed significant isoform switching. These data collectively identify a core set of changes among tissue types in a large animal model of septic-like injury.

## Results

### Intravenous infusion of LPS induces clinical and pathological signs of systemic inflammation in swine

**Fig. 1:**
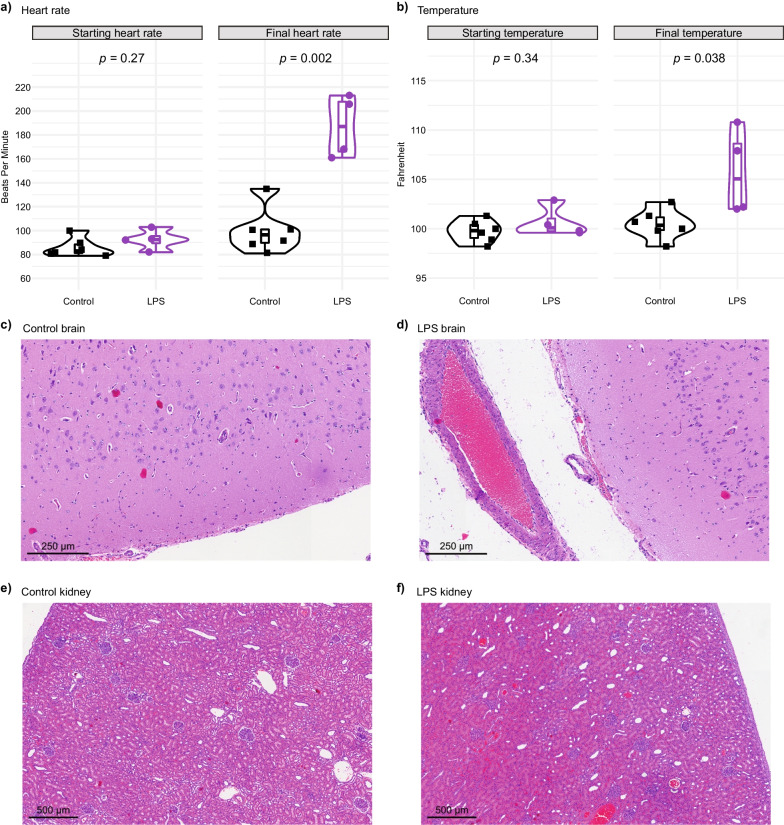
Pigs that received lipopolysaccharide (LPS) show increased heart rate, body temperature, and vascular congestion. **a** Baseline heart rate was not different between pigs assigned to control (saline) vs. LPS groups, but at the final clinical readings before killing, pigs that received LPS showed an increased heart rate *p* = 0.002. The same holds true for **b** body temperature, *p* = 0.038. Histological analysis of **c** control saline brain, **d** LPS brain, **e** control saline kidney, and **f** LPS kidney reveals significant edema and vascular congestion in the LPS tissues compared to saline controls

We administered IV the endotoxin LPS to female Yorkshire pigs (*n* = 4) compared to saline controls (*n* = 6) and monitored for clinical signs of systemic inflammation. No baseline differences were observed between groups (Additional file 2: Table S1). LPS administration resulted in elevated heart rates and body temperatures, increased arterial lactate concentration, elevated serum creatinine, and reduced urine output (Fig. 1a, b and Additional file 2: Table S1), similar to human septic cases. Pigs that received LPS demonstrated hemodynamic instability, with clinically relevant systemic hypotension defined by mean arterial pressure (MAP) less than 60 mmHg (Additional file 2: Table S1). Animals were given a constant infusion of phenylephrine and norepinephrine in escalating doses to maintain MAP equal to 60 mmHg to control hypotension (Additional file 2: Table S1). Finally, compared to saline controls, histological analysis revealed that brain and kidney tissues from LPS-injected pigs had significant edema and vascular congestion, consistent with severe sepsis in other sepsis-like models and human cases (Fig. 1c–f) [36, 37].

### Increased inflammatory response and decreased regulation of tight junctions and blood vessel pathways in brain

**Fig. 2:**
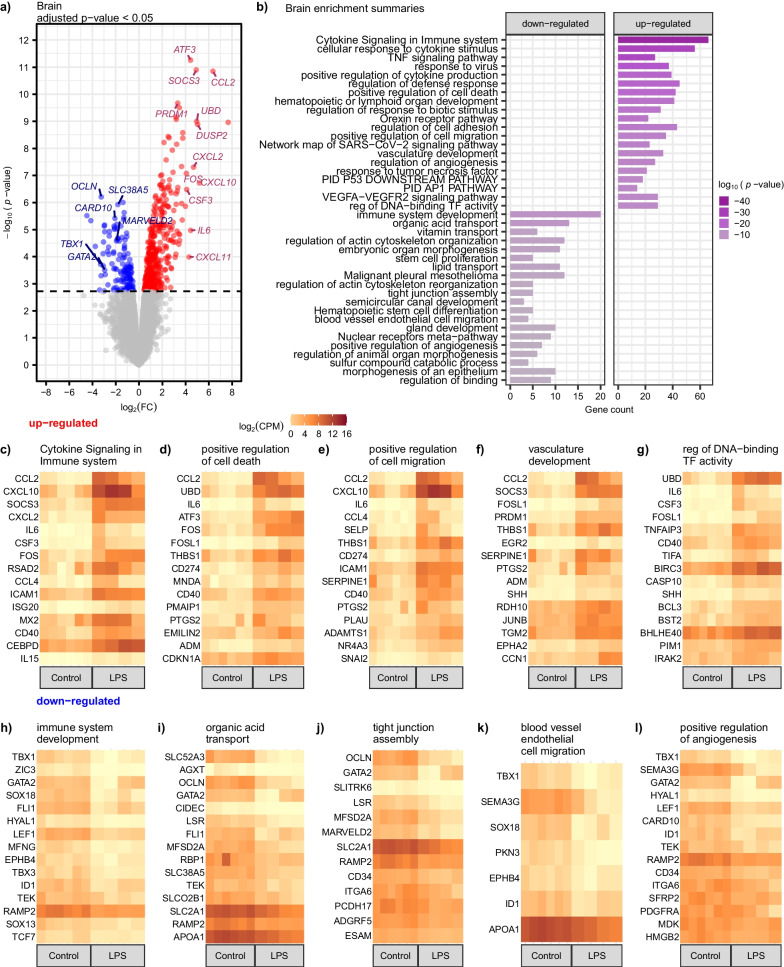
Upregulation of proinflammatory pathways and downregulation of blood–brain barrier maintenance in the brain following systemic LPS challenge. **a** Volcano plot of differentially expressed genes for LPS (*n* = 4) versus control saline (*n* = 6). Genes that are differentially expressed, with an adjusted *p* < 0.05, are indicated in blue for downregulated (log_2_ fold change < 0) and red for upregulated (log_2_ fold change > 0). Genes that are not differentially expressed, adjusted *p* $\ge$ 0.05, are shown in gray. **b** Gene ontology (GO) analysis showed an upregulation of cytokine signaling and downregulation of immune system signaling and homeostasis. The x-axis is the gene count contributing to the enrichment pathways listed on the y-axis. The color of the bar indicates the − log_10_ *p*-value. **c**–**g** Heatmaps showing gene expression, log_2_ counts per million (CPM), for each individual pig for the top upregulated genes sorted by greatest to least log_2_FC within selected enrichment functions. **h**–**l** Heatmaps showing gene expression for the top fifteen downregulated genes for each individual pig within selected enrichment functions

We identified numerous differentially expressed genes (DEGs) in the brain following LPS, with a generally stronger response in upregulated genes compared to downregulated genes (Additional file 3: Table S2). Using a conservative adjusted *p* < 0.05, we found 422 upregulated and 147 downregulated genes in the brain. Several genes had more than a 20-fold increase in expression (Fig. 2a and Additional file 3: Table S2). Activating transcription factor 3 (*ATF3*), a negative regulator of inflammation [38, 39], was among the top upregulated genes. Suppressor of Cytokine Signaling 3 (*SOCS3*) showed a greater than 30-fold increase in expression (Fig. 2a and Additional file 3: Table S2); SOCS3 downregulates cytokine signaling due to binding to both the Janus Kinase (JAK) and the cytokine receptor, which results in the inhibition of *STAT3* activation [40]. It has been shown that *SOCS3* expression is correlated with the severity of inflammation, suggesting that over-activation may be damaging [41]. Gene enrichment analysis showed significant enrichment in several inflammatory response pathways among upregulated genes and significant alteration of tight junction pathways in downregulated genes (Fig. 2b and Additional file 4: Table S3). Additional enrichment of upregulated genes was found in cytokine signaling in the immune system, positive regulation of cell death and migration, vasculature development, and regulation of DNA-binding transcription factor activity (Fig. 2c–g). Downregulated genes were enriched in pathways related to immune system development, organic acid transport, tight junction assembly, blood vessel endothelial cell migration, and positive regulation of angiogenesis (Fig. 2h–l). One of the downregulated genes in pig brains with a 4.92-fold decrease in expression was T-Box Transcription Factor 1 (*TBX1*), a transcription factor that regulates several developmental processes [42, 43]. We also found a strong downregulation of *GATA2*, a zinc finger transcription factor with known roles in the immune and hematopoietic systems [44], which showed a 7.67-fold decrease in expression. Interestingly, we found a massive 9.44 fold decrease in the level of occludin (*OCLN*), a key player in the maintenance of the blood–brain barrier [45, 46]. Previous research has identified that the blood–brain barrier is affected by sepsis and may lead to lifelong impairment among survivors [46, 47].

We additionally performed isoform-level differential expression analyses in the brain and found eight genes with multiple isoforms showing opposite expression (isoform switching) patterns (Additional file 1: Fig S1a, b) which can result in distinct biological functions. These oppositely expressed isoforms in the brain include the Proteasome 26S Subunit, ATPase 3 (*PSMC3*), a core proteasome subunit. Five isoforms of *PSMC3* are expressed in the pig brain, one isoform (ENSSSCT00000100718) is significantly upregulated with over a 30-fold increase in expression in LPS pigs compared to saline control (Additional files 1, 5 and 6: Fig S1c, Tables S4 and S5). In contrast, isoform ENSSSCT0000091007 of *PSMC3* is significantly downregulated with a 15.67-fold change decrease in expression (Additional files 1, 5 and 6: Fig S1c, Tables S4 and S5). TSC Complex Subunit 2 (*TSC2*) is another gene with multiple isoforms where not all isoforms show the same expression pattern within LPS pig brains (Additional files 1, 5 and 6: Fig S1c, Tables S4 and S5). TSC2 has been implicated to be involved in regulation of the mammalian target of rapamycin complex 1 (mTORC1) which controls cell growth [48]. It has been suggested decreased protein synthesis mediated by inhibition of mTOR (mammalian target of rapamycin) may result in sepsis-induced muscle atrophy [49]. Both *PSMC3* and *TSC2* are not called as differentially expressed when using standard gene-level analysis (see Materials and methods and Additional files 3 and 6: Tables S2 and S5), thus highlighting the importance of examining the data using different processing pipelines.

### Comparison of LPS response in pig brain significantly differs from mouse models

**Fig. 3:**
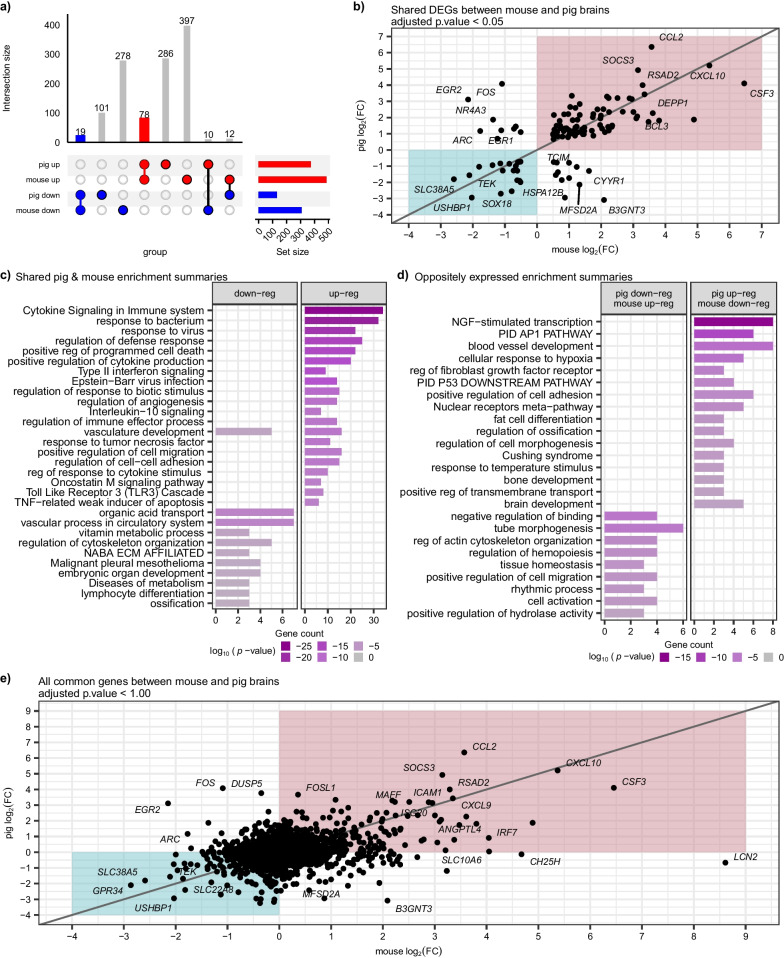
Significant differences in LPS response in the brain between pigs and mice. **a** Upset plot comparing differentially expressed genes (adjusted *p* < 0.05) in pig brain (this paper) and previously published similar mouse brain study from Kang et al. 2018 (adjusted *p* < 0.05 & absolute log_2_FC > 0.5) [17]. Rows correspond to the gene sets up or downregulated in each species. Columns indicate the intersection between those gene sets. Nineteen down and 78 upregulated genes are shared between pigs and mice. **b** Scatter plot of significantly (adjusted *p* < 0.05) differentially expressed genes common between pig and mouse. The log_2_(FC) of LPS vs. control saline genes in pig (y-axis) versus log_2_(FC) of LPS vs. control in mouse (x-axis) reveals species-specific alterations. The teal box indicates negative log_2_(FC), and the red box indicates a positive log_2_(FC) in both species. **c** Gene set enrichment analysis of genes commonly up and downregulated in pigs and mice. **d** Gene set enrichment analysis of genes oppositely regulated between pigs and mice. **e** Scatter plot of all differentially expressed genes (adjusted *p* < 1) between pig and mouse, regardless of fold change direction or significance value

We re-processed mouse LPS brain data from our previous publication to match our pig pipeline ([17], see Materials and methods). We focused our analysis on the expressed genes from both pig and mouse that had clear human orthologs (Additional file 7: Table S6). In comparing the differentially expressed genes (DEGs) found in mouse brains versus pig brains following LPS, we identified some overlapping changes but many distinct or even oppositely regulated transcripts (Fig. 3a and Additional file 7: Table S6). Although there were several hundred significantly dysregulated genes in mouse brains, there was a striking paucity of overlapping changes in LPS pig brains (Fig. 3a). To visualize this further, we plotted the log_2_ fold change of the significant DEGs from each dataset against each other (Fig. 3b). This correlation plot shows many robustly and commonly upregulated genes in both species from LPS (upper right pink quadrant in Fig. 3b) such as *CXCL10*, *CCL2*, and *CSF3,* as well as commonly downregulated genes such as *SLC38A5*, *TEK,* and *USHBP1* (lower left teal quadrant in Fig. 3b). The shared upregulated DEGs between pig and mouse brains showed strong enrichment of inflammatory pathways such as cytokine signaling, response to bacteria, defense response, etc. (Fig. 3c and Additional file 4: Table S3). Among the commonly downregulated DEGs, we found less overall enrichment but still shared responses in vascular-related pathways such as organic acid transport, vascular process/development, etc. (Fig. 3c and Additional file 4: Table S3).

However, because several DEGs were not shared between pig and mouse LPS brains, we next focused on understanding the nature of these distinct or even oppositely regulated responses (Fig. 3a, b and Additional file 7: Table S6). Of the shared significant DEGs in each species, several were significantly upregulated in pigs but significantly downregulated in mice (Fig. 3b, upper left quadrant). Notably, many of these genes were highly enriched in NGF-stimulated transcription and are known immediate-early response transcription factors, including *FOS*, *FOSB*, *JUN*, *EGR1*, *EGR2*, and *EGR3* (Fig. 3d). Conversely, several DEGs were also significantly downregulated in pigs but upregulated in mice (Fig. 3b, lower right quadrant) that showed some pathway enrichment for negative regulation of binding and tube morphogenesis (Fig. 3d). Additionally, we expanded our analysis to include all genes, regardless of significance. In this analysis, we identified numerous substantially up or downregulated genes in one species but no change or even an opposite change in the other species (Fig. 3e). One notable example is Lipocalin 2 (*LCN2)*, a gene that we previously published [17] as the single most upregulated gene and protein in mouse brains following LPS, was not significantly altered in our pig LPS data (in fact, it is slightly decreased) (Fig. 3e and Additional file 7: Table S6). Taken together, these data indicate that significant and important differences exist between rodents and our pig model of systemic inflammation, at least in the response in brain tissue.

### Substantial alternations in the kidney following LPS challenge are enriched in cytokine signaling and tube morphogenesis

**Fig. 4:**
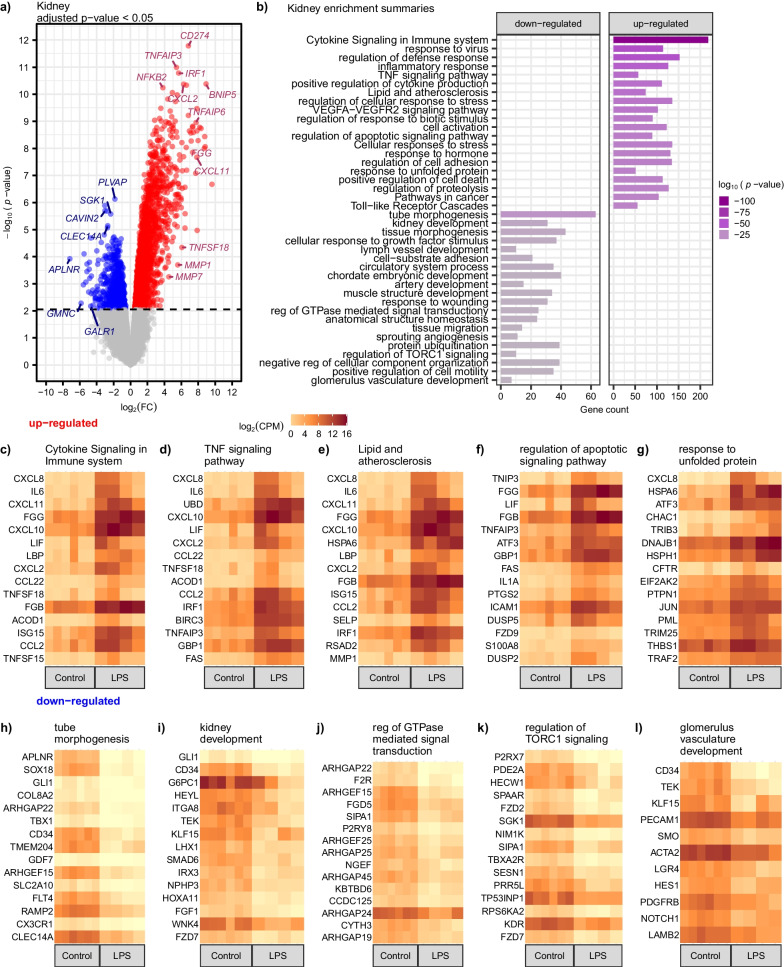
Upregulation of cytokine signaling in the immune system and downregulation of tube morphogenesis in the kidney following LPS challenge. **a** Volcano plot of differentially expressed genes (adjusted *p* < 0.05) for LPS (*n* = 4) versus control saline (*n* = 6) showed numerous differentially expressed genes. **b** Downregulated and upregulated pathways showed enrichment for cytokine signaling in the immune system, response to virus, tube morphogenesis, and kidney development, among others. **c**–**g** Heatmaps showing gene expression, log_2_ counts per million (CPM), for the top upregulated genes, sorted by greatest to least log_2_FC, within selected enrichment functions reveals potential gene targets and the corresponding pathway(s). **h**–**l** Heatmaps showing gene expression for the top downregulated genes within selected enrichment functions

We next analyzed changes that occur in kidneys in response to LPS since this organ is among the earliest impacted by septic insult. There were considerable alterations in the kidney following LPS, with 1,839 upregulated and 716 downregulated DEGs (adjusted *p* < 0.05) (Fig. 4a and Table S2). Several genes had more than a 128-fold increase in expression (Fig. 4a and Additional file 3: Table S2). *CD274* was among the most significantly upregulated genes observed in the kidney (adjusted *p* < 0.0001 and 111-fold increase in expression) (Fig. 4a and Additional file 3: Table S2). During infection or inflammation, *CD274* encodes an immune inhibitory receptor ligand that hinders T cell activation and cytokine production—an essential reaction for maintaining homeostasis of the immune response [50]. Other highly upregulated genes include TNF-$\alpha$ induced protein 3 (*TNFAIP3*), Interferon Regulatory Factor 1 (*IRF1*), and C-X-C Motif Chemokine Ligand 11 (*CXCL11*), all of which have known roles in regulating immune responses (Fig. 4b–d, f and Additional file 4: Table S3). TNFAIP3 protein is suggested to inhibit NF-kappa B activation and TNF-mediated apoptosis (Fig. 4d). The negative regulation of the NF-kappa B signaling pathway is critical for dampening excessive immune responses and, thus, tissue damage [51, 52]. IRF1 is a known transcriptional regulator and tumor suppressor involved in innate and adaptive immune responses, playing a key role in apoptosis, cell proliferation, and DNA damage response [53]. The chemokine gene *CXCL11* is fundamental to the development and function of the immune system, and higher serum levels of CXCL11 have been associated with better kidney recovery [54]. *CXCL11* is highly upregulated in the kidney following LPS challenge in our pig model, with a 222-fold increase in expression compared to saline control (Fig. 4a and Additional file 3: Table S2).

A gene set enrichment analysis of the 1839 upregulated genes further confirms an overwhelming upregulation of pathways involved in cytokine signaling in the immune system, TNF signaling pathway, and regulation of apoptotic signaling pathway (Fig. 4b–d, f and Additional file 4: Table S3). Enrichment of lipid and atherosclerosis and response to unfolded protein pathways were also observed in the kidney (Fig. 4e, g and Additional file 4: Table S3), which have been previously observed in acute inflammatory response and sepsis [55, 56]. The 716 downregulated genes in the kidney are enriched in tube morphogenesis, kidney development, GTPase-mediated signal transduction, regulation of TORC1 signaling, and glomerulus vasculature (Fig. 4a, b, h–l and Additional file 4: Table S3)—suggesting vascular functions are dysregulated in inflammation [57–59]. In summary, these data support the concept that over-activation of cytokine signaling and dysregulation of tube morphogenesis are evident in the kidney following infection and inflammatory response.

As with the brain dataset, we also performed isoform-level differential expression analyses in the kidney and observed 16 genes with multiple isoforms showing opposite expression patterns (Additional files 1, 5, 6: Fig S2a, b, Tables S4 and S5). Ankyrin Repeat And Sterile Alpha Motif Domain Containing 1A (*ANKS1A*), a regulator of ephrin receptor signaling, has five isoforms expressed in the kidney. Isoform ENSSSCT00000068005 of *ANKS1A* is upregulated with a 27-fold change increase in expression, and isoform ENSSSCT00000048052 is downregulated with a 1.87-fold decrease in expression; the remaining three other isoforms are not differentially expressed (adjusted *p* > 0.05) (Additional file 1, 5, 6: Fig S2c, Tables S4 and S5). At the standard gene-level analysis, *ANKS1A* is significantly downregulated (2.64 fold decrease in expression and adjusted *p* < 0.02) (Additional files 3, 6: Table S2 and S5). Bromodomain Containing 4 (*BRD4*) is upregulated at the gene-level analysis (2.1 fold increase in expression and adjusted *p* < 0.0024), but at the isoform level, this gene shows an up and downregulated isoform (Additional file 6: Table S5). *BRD4* is thought to be involved in regulating gene transcription by chromatin targeting, and recent reports have linked *BRD4* as a regulator of inflammation and immune response during sepsis [60]. These oppositely expressed isoforms in the kidney further demonstrate the importance of implementing multiple processing pipelines so as not to miss any potential transcriptional changes and to understand possible tissue-specific alterations better.

### Kidney and brain share a core set of transcriptional changes from LPS

**Fig. 5:**
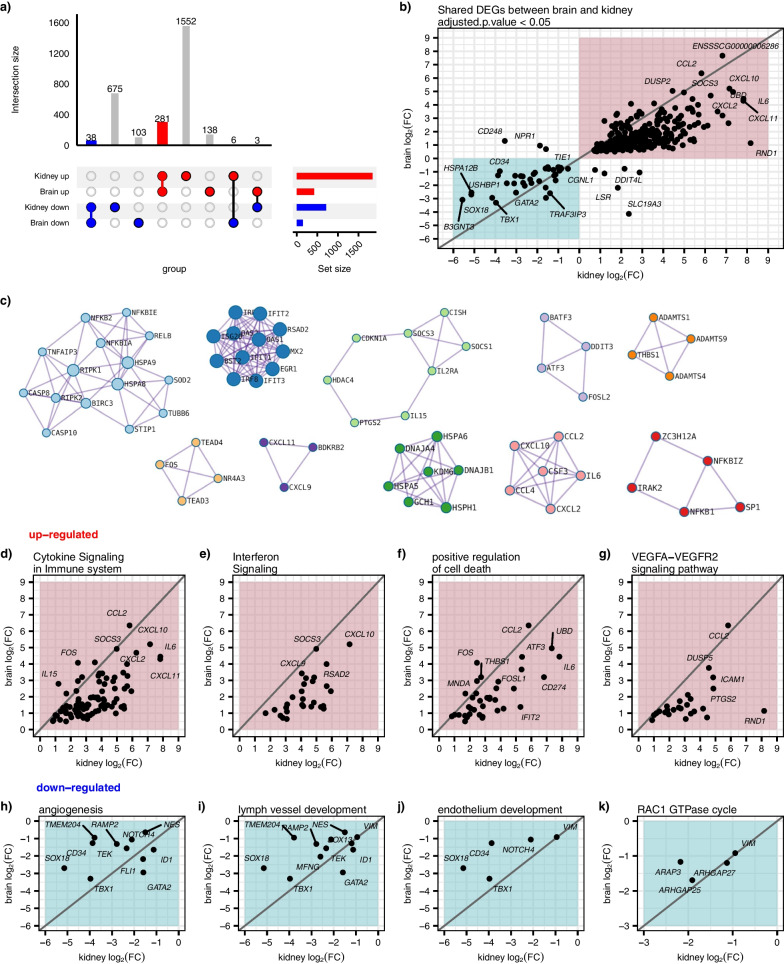
Robust transcriptional alterations and pathways are similar between the kidney and brain following LPS challenge. **a** Upset plot of commonly up and downregulated genes between the kidney and brain, with more DEG’s in the kidney than in the brain, and a few oppositely regulated genes. **b** Scatter plot of log_2_(FC) of LPS vs. control saline genes in the kidney (y-axis) versus log_2_(FC) of LPS vs. control in the brain (x-axis) shows that most of the DEG’s in the brain and kidney are in the same direction. **c** Protein–protein interaction networks demonstrates that these commonly dysregulated genes between brain and kidney likely interact functionally. **d**–**g** Scatter plot of log_2_(FC) of LPS vs. control saline genes in the kidney (y-axis) versus log_2_(FC) of LPS vs. control in the brain (x-axis) of genes enriched in upregulated GO pathways shows the kidney has the greatest transcriptional response compared to the brain for most genes. **h**–**k** Scatter plot of genes enriched in downregulated GO pathways again shows the kidney has the greatest transcriptional response compared to the brain for most genes

**Fig. 6:**
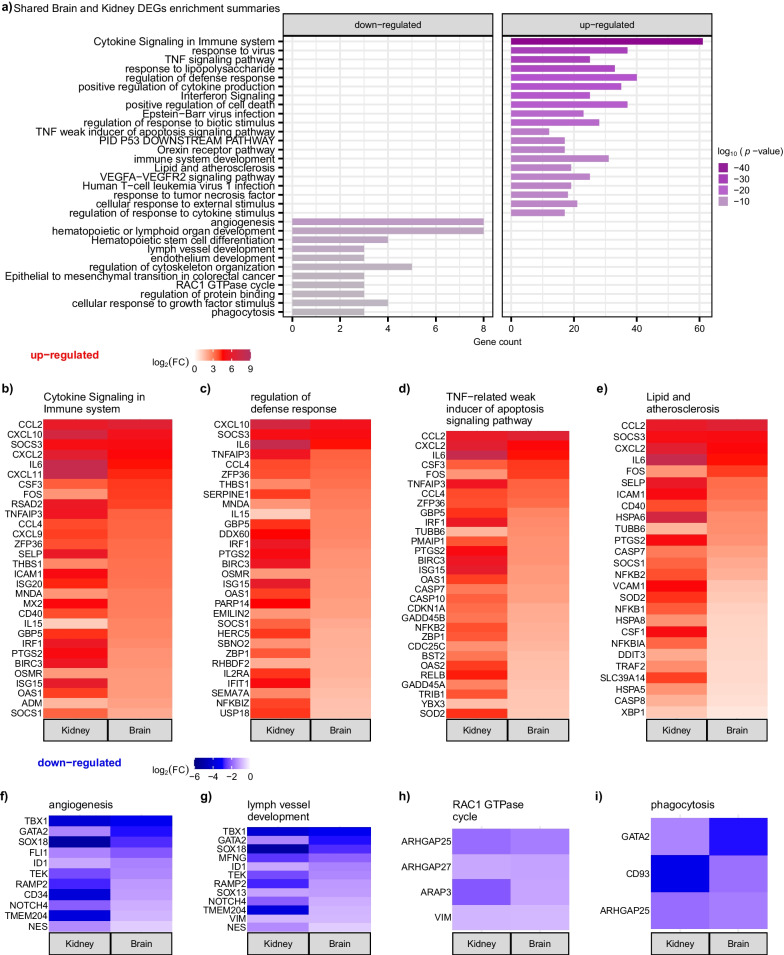
Differentially expressed genes shared between the brain and kidney after LPS show upregulation of pathogen response and downregulation of growth and development. **a** Summary of enrichment terms shared between the brain and kidney. **b**–**e** Heatmaps of the log_2_FC within the kidney and brain for selected upregulated GO terms. **f**–**i** Heatmaps of the log_2_ FC within the kidney and brain for selected downregulated GO terms

We identified more differential expressed genes (DEGs) in the kidney (2555 DEGs) than in the brain (569 DEGs), adjusted *p* < 0.05 (Fig. 5a and Additional file 3: Table S2); however, upon further investigation, most of the kidney DEGs have a similar response in the brain and vice versa (Additional file 8: Table S7). For DEGs that are common between the kidney and brain (281 upregulated and 38 downregulated, adjusted *p* < 0.05) (Additional file 9: Table S8), the kidney generally shows the greatest fold change (Fig. 5b and Additional file 8: Table S7). Several genes show similar fold changes in expression in the kidney and the brain, including upregulated genes *SOCS3*, *CXCL10*, *CCL2*, *CXCL2*, *CXCL11*, *UBD*, *IL6*, *DUSP2*, and downregulated genes *GATA2*, *TBX1*, *CD43*, *VIM*, *SOX18*, *TEK* (Fig. 5b). Genes commonly upregulated in kidney and brain tissues are enriched in cytokine signaling in the immune system, interferon signaling, positive regulation of cell death, and the VEGFA–VEGFR2 signaling pathway (Figs. 5d–g, 6 and Additional file 4: Table S3). The kidney shows the greatest fold change for upregulated genes with a few exceptions—such as *FOS* and *IL15*, which show a greater fold change response in the brain than in the kidney (Fig. 5d and Additional file 8: Table S7). Fos Proto-Oncogene, AP-1 Transcription Factor Subunit (*FOS*) is thought to regulate cell proliferation, differentiation, and transformation, and increased expression of *Fos* in a rat model has been suggested to mediate the release of norepinephrine which may impact limbic and hypothalamic function [61]. Interleukin 15 (*IL15*) also shows a greater fold change response in the brain than in the kidney (Fig. 5d and Additional file 8: Table S7). IL15 is a cytokine that regulates T and natural killer cell proliferation and activation; it has been suggested that *IL15* becomes upregulated after neuroinflammation [62]. IL15 may modulate gamma-aminobutyric acid (GABA), a neurotransmitter inhibitory and serotonin transmission, ultimately disrupting anxiety, mood, sleep, and memory [62]. Thirty-eight genes are commonly downregulated in the kidney and the brain following LPS (Fig. 5a, b and Additional file 9: Table S8). These genes are enriched in angiogenesis, lymph vessel development, endothelium development, and RAC1 GTPase cycle (Figs. 5h–k, 6 and Additional file 4: Table S3). Again, the kidney shows the greatest absolute fold change compared to the brain, with a few exceptions, including *GATA2* (Fig. 5h–k). As noted earlier, *GATA2* has known roles in the immune and hematopoietic systems [44] and is strongly downregulated in the LPS pig brains (7.67-fold decrease in expression, Additional file 3: Table S2). Very few genes (nine total) had an opposite expression pattern between the kidneys and the brain (Fig. 5a and Additional file 8: Table S7). In summary, there are numerous transcriptional alterations following LPS challenge, and a core set of these alterations are common between the brain and kidney, suggesting a shared response among organs.

### Robust transcriptional alterations in the blood following LPS

Blood showed numerous transcriptional alterations with 2192 upregulated and 1295 downregulated genes in LPS compared to saline control pigs (Additional files 1, 3: Fig S3 and Table S2). Upregulated genes are involved in cytokine signaling in the immune system, cellular responses to stress, cell cycle checkpoints, signaling by Rho/Miro GTPases & *RHOBTB3*, and apoptosis. Downregulated genes are involved in neutrophil degranulation, signaling by Rho GTPases, regulation of vesicle-mediated transport, inflammatory response, and small GTPase-mediated signal transduction (Additional files 1, 4: Fig S3b and Table S3). Interestingly genes involved in signaling by Rho/Miro GTPases and *RHOBTB3* are upregulated, while another set of genes involved in signaling by Rho GTPases and small GTPase-mediated signal transduction are downregulated (Additional files 1, 4: Fig S3 and Table S3). Bacterial toxins interfere with RhoGTPases resulting in modifications of epithelial and endothelial barriers that contribute to the dispersal of bacteria within the host [63, 64]. Rho protein inhibition or activation has been associated with sepsis dysfunction according to the cellular system being evaluated [64].

We additionally performed isoform-level differential expression analyses in the blood and observed 53 genes with multiple isoforms showing opposite expression patterns (Additional files 1, 5 and 6: Fig S4a, b, Tables S4 and S5). Rho Guanine Nucleotide Exchange Factor 10-Like Protein (*ARHGEF10L*) belongs to the RhoGEF subfamily and is involved in signal transduction. In the blood, *ARHGEF10L* has three isoforms expressed, of which one is downregulated with a sevenfold decrease in expression, and the other two isoforms are upregulated with a 21- and 2.7-fold increase in expression compared to saline control (Additional files 1, 5, 6: Fig S4a, b, Tables S4 and S5). *ARHGEF10L* is not differentially expressed at the gene level (Additional file 6: Table S5). C–C Motif Chemokine Receptor 2 (*CCR2*) protein is a receptor for monocyte chemoattractant protein-1, which may play a role in monocyte infiltration [65, 66]. Within pig blood, *CCR2* expresses four isoforms, two of which are significantly upregulated, and the other two are significantly downregulated (Additional files 1, 6: Fig S4d and Table S5). Interestingly, *CCR2* at the gene level is significantly downregulated (12-fold decrease and adjusted *p* < 0.0005) (Additional file 6: Table S5). In summary, numerous oppositely expressed isoforms are observed in the blood, further demonstrating that a gene-level only analysis is inadequate to capture the transcriptional expression patterns following LPS exposure.

### Consistent upregulation of genes involved in NF-kappaB signaling and downregulation of genes involved in RHO GTPase cycle within pigs that received LPS, regardless of tissue type.

A comparison of DEGs among the brain, kidney, and blood show 149 upregulated and 11 downregulated genes that are common among all three tissues (Additional files 1, 9: Fig S5 and Table S8). The 149 commonly upregulated genes are significantly enriched in cytokine signaling, TNF signaling pathway, PID IL12 2PATHWAY, and regulation of I-kappaB kinases/NF-kappaB signaling (Additional files 1, 4: Fig S5b, d–g and Table S3). Nuclear factor kappa B (NF-κB) is a transcription factor [67] that regulates the expression of inflammatory mediators and defensive response [68, 69]. Upregulation of NF-kappaB has been noted to occur within septic individuals, and over-activation has been associated with poorer clinical outcomes and a higher mortality rate [70]. Upregulation of I-kappaB kinases/NF-kappaB signaling is observed within the brain, kidney, and blood of our LPS pig model. Furthermore, we observed eleven genes that are commonly downregulated among the brain, kidney, and blood, of which three genes (*ARHGAP25*, *VIM*, and *ARAP3*) are involved in the RHO GTPase cycle—indicating a general response to bacterial toxins that could be used as potential targets for therapies.

## Discussion

Sepsis results from an exaggerated inflammatory response due to a known or suspected pathogen. When left unchecked, the cytokine storm and release of proinflammatory mediators are harmful to the host and lead to acute and chronic damage to several organs, including the brain [6]. Though sepsis can impact individuals of all ages, the majority of individuals are elderly. Survivors of sepsis, in particular the elderly, are at high risk for lasting organ dysfunction. The kidney is among the earliest organs impacted [71], and sepsis commonly results in acute kidney injury (AKI) and the development of chronic kidney disease in survivors [9]. Sepsis survivors are additionally at high risk of new onset cognitive and behavioral dysfunction [4, 15, 16, 24]. We set out to identify early tissue-level changes that arise subsequent to LPS administration and promotion of a highly dysregulated immune response with the goal to better understand the transcriptional cascades that occur and may be targeted to protect and preserve organ functions. The National Institute of Health (NIH), specifically the National Institute of General Medical Sciences (NIGMS), has encouraged the use of novel sepsis models to help discern pathways that are associated with the pathophysiology and resolution of sepsis in humans (https://grants.nih.gov/grants/guide/notice-files/NOT-GM-19-054.html). We chose the Yorkshire pig as this large (70–74 kg, Additional file 2: Table S1) animal model shares closer physiological and anatomical similarities to humans compared to rodents.

The pathophysiology of acute kidney injury (AKI) in almost any setting is complex. Sepsis-induced AKI can, in part, occur secondary to sepsis-induced hemodynamic instability. Hemodynamic instability, as traditionally defined, may or may not be present in the setting of sepsis initially. In the experiment presented here, LPS was infused slowly and produced a clinical syndrome in which multi-organ system failure occurred, leading inevitably to acute kidney injury and other organ injuries despite maintaining systemic blood pressure via pressor agents (see Materials and Methods and Additional file 2: Table S1). We sought to assess the early transcriptional alterations in the kidney, brain, and blood following LPS challenge to better understand multi-organ failure in a large animal model of systemic inflammation.

We identified robust transcriptional alterations in the brain, kidney, and whole blood following LPS. In the brain, upregulated responses are highly enriched in inflammatory pathways, and downregulated responses are enriched in tight junction and blood vessel functions. Substantial changes also occurred in kidneys following LPS with several enriched pathways for upregulated gene sets (cytokines, lipids, unfolded protein response, etc.) and downregulated gene sets (tube morphogenesis, glomerulus development, GTPase signal transduction, etc.). We also found significant dysregulation of genes in whole blood that fell into several gene ontology categories (cytokines, cell cycle, neutrophil degranulation, etc.). We observed a very strong correlation in the responses between the brain and kidney, with significantly shared pathways in both upregulated genes (cytokine signaling, cell death, and VEGFA pathways) as well as downregulated genes (vasculature and RAC1 GTPases). In brief, we have identified a core set of shared genes and pathways in a pig LPS-induced systemic inflammation model.

We found several interesting isoform switching events in all tissues profiled. The majority of transcriptome analyses compresses all transcript-level changes into a single isoform for any one gene. The conventional gene-level analysis did not detect most of the genes with significant isoform switching. Low expression levels of isoforms or inverse changes in other isoforms of the same gene, canceling the net change at the gene expression level, may explain why these differentially expressed isoforms are missed by standard gene-level analysis. Our isoform-level analysis revealed several instances of only a single isoform changing or even oppositely regulated isoforms for the same gene. Another interesting finding was that isoform switching showed substantial heterogeneity across the brain, kidney, and whole blood, suggesting a tissue-specific isoform response. The choice of methodology plays an important role in assessing transcriptional alterations. The gene-level limma/voom method operates with summarized gene-level counts, focusing on the expression of entire genes rather than individual isoforms [72]. Consequently, it is not suitable for evaluating isoform-level differences. In contrast, the Sleuth pipeline was purposefully designed to assess isoform differences and necessitates isoform-level quantification as input to determine differential expression [73]. Our decision to employ both of these independent workflows provided several significant insights. Firstly, it enabled us to identify a set of transcripts that consistently exhibited differential expression, irrespective of the choice of alignment and statistical analysis methods. Secondly, our approach underscored the critical importance of evaluating isoform-level differences, as some genes might be overlooked when solely utilizing a gene-level approach. Lastly, our findings emphasize the need for researchers to consider the isoforms of a gene when expanding upon the results presented in this study, particularly when pursuing the identification of potential biomarkers. This consideration should encompass an assessment of how various isoforms are expressed in different tissue types, recognizing the potential impact of isoform diversity on the overall understanding of gene function and regulation.

Comparison of the LPS-induced response in the pig brain to data in a similar mouse model demonstrated some overlapping changes and gene sets but also numerous striking differences. One notable example is Lipocalin 2 (*LCN2)* which we previously identified as the most upregulated transcript and protein in mouse brains following LPS exposure regardless of the sex of the mouse [17], but this gene was unaltered in pig brains from LPS. There were hundreds of genes uniquely altered in pigs compared to mice. Moreover, of the shared significant DEGs in each species, several were oppositely regulated, and many of these genes were highly enriched in NGF-stimulated transcription and are known immediate-early response transcription factors, including *FOS*, *FOSB*, *JUN*, *EGR1*, *EGR2*, and *EGR3*. Though these are not perfectly comparable datasets in terms of dose, timing, and sex, this analysis does indicate a fundamentally different tissue-level response to LPS, at least in brain tissue.

In summary, we have identified several robust and shared transcriptional changes that occur in a large animal model of systemic inflammation, with a specific focus on the brain, kidney, and blood. Although blood can be profiled in human patients at varying stages of sepsis, capturing the tissue-level changes that occur early in the septic response would be extraordinarily difficult or even impossible. Our study is not without limitations as we utilized only female pigs for safety reasons, and only a limited number of animals were profiled though we still uncovered highly significant and consistent transcriptomic alterations. These data nominate several important molecular players that are dysregulated in systemic inflammation and can be the subject of further inquiry as biomarkers or targeted therapeutics.

## Materials and methods

### In vivo LPS administration

Outbred Yorkshire (*Sus scrofa*) female swine were injected intravenously with the endotoxin lipopolysaccharide (LPS/*n* = 4) or saline (control/*n* = 6). Pigs were aged 5 months and weighed 70–74 kg, a size that is comparable to humans. Given the size, the Mayo Clinic animal facility could not house fully adult male swine as they are large and can be aggressive to the point of being unsafe to staff; thus, young adult females were used. Animals had access to water, were fed a diet of Purina Lab Porcine Grower Diet 5084, and were group housed. Pigs received a five-step anesthetized administration process of telazol, xylazine, glycopyrrolate, and plasmalyte, followed by LPS or saline injection rates 0.50–1.00 mL/h (Additional file 2: Table S1). Cannulas were placed surgically in the jugular vein and carotid artery for pathogen infusion and clinical monitoring. A continuous intravenous infusion of LPS (Escherichia coli LPS 026:B6) at 2 μg/kg/h was initiated. The duration measured from injection to killing ranged from 190 to 562 min (Additional files 2, 10: Tables S1 and S9). Animals were monitored for vital signs including heart rate, respiration rate, oxygen saturation (SPO2), blood pressure, mean arterial pressure, body temperature, and carbon dioxide (Additional files 2, 10: Tables S1 and S9). Urine output rate (via bladder catheter) and serum creatinine (via femoral artery) measures were performed (Additional files 2, 10: Table S1 and S9). Before LPS or saline administration, three serum creatinine measures were obtained (baseline serum creatinine). Following LPS or saline administration, urine output rate and serum creatinine were measured every 20 min until the end of the experiment (Additional file 2: Table S1). Designated by the veterinarian, a priori termination humane endpoints were excessive or uncontrolled bleeding or uncorrectable clinical deterioration as evidenced by dramatic changes in vital signs or cardiac rhythms (Additional files 2, 10 Tables S1 and S9). Euthanasia was initiated upon the occurrence of multi-organ dysfunction unresponsive to supportive measures. We monitored for indications such as vascular collapse (MAP < 60), heart rate exceeding 60 beats per minute, or absence of urine output at three time points. The LPS-treated pigs varied in their individual euthanasia time points due to individual pig variation of multi-organ dysfunction. In contrast, all saline control pigs were euthanized at 300 min as they did not exhibit any organ dysfunction. Blood samples were collected using EDTA tubes. Whole blood samples were frozen at − 80 °C. Brain (prefrontal cortex) and kidney (medulla and cortex) samples were collected immediately after death and frozen at − 80 °C for sequencing experiments.

### Histological evaluation

The brain was excised, and tissue was collected for histologic analysis. The tissue was fixed in neutral-buffered 10% formalin, embedded in paraffin wax, and sectioned at 4 µm. Sections were mounted on glass slides and stained with H&E stain. Tissues were reviewed by a board-certified veterinary pathologist (NMG).

### Bulk RNA-sequencing (RNAseq) library construction and sequencing

We collected prefrontal brain cortex, kidney, and whole blood from pigs after IV administration of LPS (*n* = 4) compared to saline controls (*n* = 6); for a total of 30 RNAseq samples. In 5-mL Eppendorf tubes, 100 mg of each sample received 500 μL of RNAlater (Invitrogen cat # AM7021) and were stored at – 80 ℃. Frozen brain and kidney samples were dissociated using PowerBead Tubes (Qiagen cat # 13114–50) in a Qiagen TissueLyser at full speed for three minutes. Total RNA was isolated from homogenized brain and kidney samples using Qiagen’s RNeasy Mini Kit (cat # 74104). RNA was extracted from blood samples using the Paxgene Blood RNA protocol CMG1084 on a Chemagic 360 instrument (PerkinElmer cat # 2024-0020). Approximately 200 ng of total RNA was used for mRNA purification and library preparation using the Kapa mRNA Hyper Prep Kit (cat # KR1352). 1.2 nM cDNA was loaded onto Illumina NovaSeq 6000 S4 flow cells for each sample. Samples were sequenced over three runs to 50 million (M) 2 × 100 base paired-end reads and demultiplexed by the Yale School of Medicine sequencing core. Some samples had a low number of sequences and thus were re-sequenced, resulting in technical replicates (Additional file 2: Table S1).

### Read quality control

Fastq files were checked for quality using FastQC version 0.11.9 [74] and aggregated using MutliQC version 1.10.1 [75]. Raw RNA-seq reads were trimmed for quality and removed adapter content using BBDuk as part of the BBMap package version 38.90 [76]. The TruSeq single read 1 i7_Illumina_UDI_10nt_index & read 2 i5_Illumina_UDI_10nt_index adapter content was removed by specifying *ref* = *adapter.fa*. Trimming was accomplished starting from the 3’ end, *ktrim* = *r*, and implementing a length of 23-mers to trim both paired reads to the same length. A 23-11-mer, *mink* = *11*, was used to look for shorter kmers at the ends, and *hdist* = *1* was used to allow one mismatch. Finally, Bushnell, 2014 recommended adding the tbo flag for paired-end fragment libraries, which trims adapters based on pair overlap detected using BBMerge, part of the bbduk BBMap package [76]. Post-trimming quality was checked using FastQC [74] and MultiQC [75].

### Reference genome and transcriptome assembly

The top-level Ensembl *Sus scrofa* reference genome version 11.1 was downloaded from ensembl.org, Sus_scrofa.Sscrofa11.1.dna.toplevel.fa version 107 [77]. The reference genome includes 1–18 autosomes, mtDNA, the X chromosome, the Y chromosome, and contigs. All pigs in this study are reported as (38, XX) female and do not contain a Y chromosome. To avoid mis-mapping of homologous X–Y sequence reads, it has been shown that aligning samples that do not contain a Y chromosome to a Y-masked reference genome improves expression estimates on the X chromosome [78]. We hard-masked the Y chromosome by changing all Y chromosome nucleotides [ATGC] to N using a *re.sub* command in a custom python script (see GitHub page https://github.com/fryerlab/LPS_pigs). After creating the Y-masked *Sus scrofa* reference genome, we indexed the reference genome using STAR version 2.7.8a [79] with the option –genomeFastaFiles to indicate the reference genome, Sus_scrofa.Sscrofa11.1.dna.toplevel.Ymask.fa and –sjbdGTFfile to indicate the gene annotation file, Sus_scrofa.Sscrofa11.1.107.gtf improve alignment among known splice junctions [79]. The above was repeated for the reference transcriptome, Sus_scrofa.Sscrofa11.1.cdna.all.fa, version 107 for pseudo-alignment of RNAseq reads implementing Kallisto version 0.46.2 with default parameters [80].

### RNAseq alignment and gene-level expression quantification

Quantification estimates for each sample were obtained using STAR version 2.7.8a [79] following the twopassMode alignment and quantMode to get the read counts for each geneID. Each sample's resulting geneID-level count file was combined into a matrix of counts where each row represents a geneID, and each column is a sample.

### Isoform quantification estimates

In addition to gene-level quantification, transcript-level estimates were obtained using Kallisto version 0.46.2 [80]. RNAseq reads will often align to multiple transcripts of the same gene, and thus multi mapping of reads is part of the Kallisto algorithm to properly determine the abundances of gene isoforms [80]. Transcript-level qualification estimates per sample were obtained from running Kallisto quant with the following parameters: *–bias* to correct abundance estimates from a learned parameter model of the sequence bias in the data, and *-b 25* to specify the number of bootstrap quantification estimates to perform.

### Quantifying technical and biological variation in RNA-seq expression data

Utilizing variancePartition version 1.20.0, a linear mixed model was employed to quantify transcriptome expression variation that can be explained by a trait attribute [81]. Variation within-group (LPS or control), weight (kg), minutes from injection till killing (mins), age (days), and dose (mL/h) were examined for each tissue. We conducted a comparative analysis between the control group and LPS-treated pigs, examining various parameters, including body weight, age, dose, heart rate, temperature, and time from injection to killing. To assess the differences, we utilized either a two-sample t-test or a Wilcoxon rank-sum test, when appropriate (Additional file 2: Table S1). Specifically, a two-sample t-test was applied when the assumptions of both homogeneity of variance and normality were met. We formally evaluated these assumptions using Levene’s test for homogeneity of variance and the Shapiro–Wilk test for normality. In cases where either the assumption of homogeneity of variance or normality was not satisfied, we employed the Wilcoxon rank-sum test to determine differences in the distributions between the saline and LPS-treated pigs. We utilized both a t-test and a Wilcoxon rank-sum test to compare the control and LPS-treated pigs across the various clinical parameters. The *p*-values obtained from these tests closely resembled each other, indicating that the choice of statistical test in this context has minimal influence on the resulting conclusions of the analysis (see Additional file 2: Table S1).

### Inference of differential gene expression

Gene-level differential expression analysis between control (*n* = 6) and LPS (*n* = 4) pigs for frontal brain cortex, kidney, and whole blood was performed using the limma/voom pipeline [72, 82]. Using the DGEList function in the limma package, the counts matrix, annotation information, and the clinical attributes for each sample were read into R. Technical replicates from within a tissue were summed together using sumTechReps function in edgeR version 3.30.3 [83]. Counts were then normalized for library size differences using counts per million (cpm) and log-transformed (lcpm) part of the edgeR package [83]. Next, the normalized counts were filtered to remove lowly expressed genes using the function filterByExpr [84]; see Additional file 11: Table S10 for the filtered gene-level counts table for each tissue (Additional file 11: Table S10). Normalization factors were calculated using the calcNormFactors function with the trimmed mean of M-values method (TMM) [83, 85]. The counts were then voom transformed using the function voomWithQualityWeights, which combines observational-level weights with estimated sample-specific weights [72, 82, 86]. A model was created to compare LPS and saline where each coefficient corresponds to a group mean and minutes from injection till the killing was added as a covariate. Genes were deemed differentially expressed between control and LPS pigs when the adjusted *p* < 0.05 using the Benjamini–Hochberg false discovery rate (FDR) method [82] (Additional file 3: Table S2). The term “adjusted *p*-value” is utilized throughout the paper, following the toptable definition in the limma package [82]. This definition clarifies that the adjusted p-values represent bounds on the FDR, not conventional p-values associated with significance levels, and are sometimes referred to as "q-values", directly related to FDR [82].

### Isoform differential expression analysis

Isoform-level differential expression analysis between control (*n* = 6) and LPS (*n* = 4) pigs for frontal brain cortex, kidney, and whole blood was performed using the Sleuth pipeline with the Kallisto bootstrap quantification estimates [73]. Sleuth models the technical variability using the bootstraps calculated from Kallisto to distinguish technical variability from biological variability. The observed abundance estimates are the sum of counts and the technical noise; see Additional file 12: Table S11 for transcript-level counts data used for the Sleuth differential expression analysis (Additional file 12: Table S11). Sleuth estimates the biological variance when determining whether transcripts are differentially expressed using the variance from the technical and biological estimates [73]. A model was created to compare LPS and saline; minutes from injection till the killing was added as a covariate. Transcripts are defined as being differentially expressed between control and LPS pigs when the Wald test adjusted *p*-value is < 0.05 using a Benjamini–Hochberg false discovery rate [73, 82] (Additional file 5: Table S4).

### Gene function and enrichment analysis

We examined gene enrichment terms from the differentially expressed genes for the brain, kidney, and whole blood (Additional file 4: Table S3). We used the Metascape web tool, which utilizes a hypergeometric distribution to identify enriched gene ontology (GO) terms [87], with an adjusted Fisher exact *p*-value cutoff < 0.05 to select significantly enriched terms.

### Reprocessing of previously published LPS mouse data

We obtained RPKM (Reads Per Kilobase Million) counts data from previously published male mouse brain data for saline control (*n* = 4) and LPS challenge (*n* = 4) [17]. RPKM values were log-transformed to counts per million (lcpm) part of the edgeR package [83]. Similar to the pig data, the normalized counts were filtered to remove lowly expressed genes using the function filterByExpr [84]. Normalization factors were calculated with the trimmed mean of M-values method (TMM) [83, 85]. Counts were voom transformed, and a model was created to compare LPS and saline. Genes were defined as being differentially expressed between the control and LPS mice when the adjusted *p* is $<$ 0.05 using a Benjamini–Hochberg false discovery rate and an absolute log_2_FC > 0.5 (Additional file 7: Table S6).

### ARRIVE guideline compliance

The project is in accordance with the Animal Research: Reporting of In Vivo Experiments (ARRIVE) guidelines to help aid in transparent reporting. The study design compares pigs that received LPS (*n* = 4) to pigs that received saline (*n* = 6). Only female pigs were used in the experiment as male pigs are too aggressive. Pigs were randomly selected to receive either LPS or saline. Experimenters were not blinded to the group allocation. Pig vitals were measured throughout the experiment, and comparisons between saline and LPS were noted (Additional file 2: Table S1). Statistical methods are described in detail under various Materials and Methods sections; specific software information and reproducible code are available on the git repository; https://github.com/fryerlab/LPS_pigs.

## Significance

Sepsis results from an overwhelming immune response to a pathogen and is a tremendous clinical problem with nearly 1–3 million new cases each year in the US alone. We have good treatments that clear the pathogen, but very few therapies exist to prevent long-term organ damage, primarily because we lack an understanding of the tissue-level changes that occur during the initial stages of inflammation. Here, we modeled the acute inflammatory aspects of systemic inflammation by administering the endotoxin lipopolysaccharide (LPS) to Yorkshire pigs and assessing responses in brain, kidney, and blood with RNAseq and have identified commonly dysregulated genes and pathways.

### Supplementary Information

**Additional file 1: Fig S1.** Isoform-specific alterations in the brain reveal eight genes with opposite expression patterns. **Fig S2.** Multiple gene isoforms show opposite expression patterns in the kidney and are involved in neuron remodeling. **Fig S3.** Genes involved in the signaling by Rho GTPases are upregulated and downregulated in the blood of LPS challenged pigs. **Fig S4.** Numerous isoform alterations in the blood following LPS challenge. **Fig S5.** Differentially expressed genes shared among the brain, kidney, and blood reveal consistent upregulation of cytokine signaling, TNF signaling pathway, and PID IL12 2PATHWAY, and downregulation of genes involved in the RHO GTPase cycle following LPS challenge.

**Additional file 2: Table S1.** Clinical differences between control saline and LPS-treated pigs. Clinical data for each pig sample. Clinical data include weight, age, dose, the timing of the experiment, and vital signs

**Additional file 3: Table S2.** Differentially expressed genes by RNAseq after LPS challenge in pigs for brain, kidney, and blood. Differentially expressed genes in the brain, kidney, and blood, adjusted *p* < 1.00 following processing with the star/limma-voom gene-level pipeline (see Materials and methods). Each tab contains the genes that met the minimum expression threshold for that tissue and are included in the differential expression analysis. Genes are considered differentially expressed if the adjusted *p*-value is < 0.05.

**Additional file 4: Table S3.** Metascape.org gene ontology enrichment analysis for up and downregulated genes in the brain, kidney, and blood following LPS challenge. Enrichment analysis was performed individually for up and downregulated genes within each tissue utilizing Metascape.org. Enriched GO terms are clustered into summaries. Log p-value and q-value are reported for each term, along with the corresponding gene symbols within each term

**Additional file 5: Table S4.** Differentially expressed isoforms after LPS challenge in pigs by RNAseq for brain, kidney, and blood. Differentially expressed isoforms in the brain, kidney, and blood, adjusted *p* < 1.00 following the kallisto/sleuth isoform-level pipeline (see Materials and methods). Each tab contains the isoforms that met the minimum expression threshold for that tissue and are included in the differential expression analysis. Isoforms are considered differentially expressed if the adjusted *p* < 0.05

**Additional file 6: Table S5.** Isoforms of the same protein-coding gene show opposite expression patterns. Showing results for when there are at least two isoforms for a gene and one isoform is reportedly upregulated (log_2_FC > 0 & adjusted-*p* < 0.05), while at least one other isoform is reportedly downregulated (log_2_FC < 0 & adjusted-*p* < 0.05).

**Additional file 7: Table S6.** Differentially expressed genes between mouse and pig brains following LPS challenge reveal oppositely expressed genes between species. Inference to determine if genes are consistently up or downregulated among the pig and mouse species challenged with LPS compared to saline controls. To compare which differentially expressed genes are shared or unique between pig and mouse, the gene ids were first converted to human gene symbols following gprofiler2 gorth function (see Materials and methods)

**Additional file 8: Table S7.** Analysis of differentially expressed genes between tissues reveals largely consistent expression patterns of genes among tissues and a few tissue specific gene expression alterations. Inference of differentially expressed genes between tissues to determine if genes are consistently up or downregulated or show an opposite expression pattern between tissues.

**Additional file 9: Table S8. **Differentially expressed genes shared and unique among the brain, kidney, and blood. Differentially expressed genes, adjusted *p* < 0.05, which are shared and unique among the brain, kidney, and blood following LPS challenge. Showing results for the samples were processed following the star/limma-voom pipeline.

**Additional file 10: Table S9.** Sample information. Sample information including sample ID, group (control or LPS), weight, age, and various start and end clinical values.

**Additional file 11: Table S10.** Gene-level counts per million (CPM) data for each tissue. Filtered to remove lowly expressed and keep only protein coding genes, gene-level counts per million (CPM) data for each tissue. This is the counts data used for the gene-level differential expression analysis with limma/voom pipeline.

**Additional file 12: Table S11.** Isoform-level transcripts per million (TPM) data for each tissue. Filtered to remove lowly expressed and keep only protein coding transcripts, isoform-level transcripts per million (TPM) data for each tissue. This is the counts data used for the isoform-level differential expression analysis with Kallisto/Sleuth pipeline.

## Data Availability

The datasets generated during this study are available at SRA BioProject accession PRJNA723823. Scripts to reproduce the analysis and figures presented in this paper are available at GitHub: https://github.com/fryerlab/LPS_pigs. Interactive apps to examine differentially expressed genes and correlation of the log_2_FC between tissues and species: https://fryerlab.shinyapps.io/LPS_pigs/

## Abbreviations

AKI: Acute kidney injury
CNS: Central nervous system
DEG: Differentially expressed gene
LPS: Lipopolysaccharide
IV: Intravenous
GO: Gene ontology
SIRS: Systemic inflammatory response syndrome
