# Supplementary material for: Commonly disrupted pathways in brain and kidney in a pig model of systemic endotoxemia
Source: J Neuroinflammation. 2024 Jan 4;21:9. doi: 10.1186/s12974-023-03002-6 (PMC10765757; doi:10.1186/s12974-023-03002-6)
Supplement: Supplementary file 1 — Additional file 1: Fig S1. Isoform-specific alterations in the brain reveal eight genes with opposite expression patterns. Fig S2. Multiple gene isoforms show opposite expression patterns in the kidney and are involved in neuron remodeling. Fig S3. Genes involved in the signaling by Rho GTPases are upregulated and downregulated in the blood of LPS challenged pigs. Fig S4. Numerous isoform alterations in the blood following LPS challenge. Fig S5. Differentially expressed genes shared among the brain, kidney, and blood reveal consistent upregulation of cytokine signaling, TNF signaling pathway, and PID IL12 2PATHWAY, and downregulation of genes involved in the RHO GTPase cycle following LPS challenge. [file 12974_2023_3002_MOESM1_ESM.docx]

## ADDITIONAL FILE TABLES

### Table S1. Clinical differences between control saline and LPS-treated pigs.

Clinical data for each pig sample. Clinical data include weight, age, dose, the timing of the experiment, and vital signs.

### Table S2. Differentially expressed genes by RNAseq after LPS challenge in pigs for brain, kidney, and blood.

Differentially expressed genes in the brain, kidney, and blood, adjusted p-value < 1.00 following processing with the star/limma-voom gene-level pipeline (see Materials and methods). Each tab contains the genes that met the minimum expression threshold for that tissue and are included in the differential expression analysis. Genes are considered differentially expressed if the adjusted p-value is < 0.05.

### Table S3. Metascape.org gene ontology enrichment analysis for up and down-regulated genes in the brain, kidney, and blood following LPS challenge.

Enrichment analysis was performed individually for up and down-regulated genes within each tissue utilizing Metascape.org. Enriched GO terms are clustered into summaries. Log p-value and q-value are reported for each term, along with the corresponding gene symbols within each term.

### Table S4. Differentially expressed isoforms after LPS challenge in pigs by RNAseq for brain, kidney, and blood.

Differentially expressed isoforms in the brain, kidney, and blood, adjusted p-value < 1.00 following the kallisto/sleuth isoform-level pipeline (see Materials and methods). Each tab contains the isoforms that met the minimum expression threshold for that tissue and are included in the differential expression analysis. Isoforms are considered differentially expressed if the adjusted p-value < 0.05.

### Table S5. Isoforms of the same protein-coding gene show opposite expression patterns.

Showing results for when there are at least two isoforms for a gene and one isoform is reportedly up-regulated (log_2_FC > 0 & adjusted-p-value < 0.05), while at least one other isoform is reportedly down-regulated (log_2_FC < 0 & adjusted-p-value < 0.05).

### Table S6. Differentially expressed genes between mouse and pig brains following LPS challenge reveal oppositely expressed genes between species.

Inference to determine if genes are consistently up or down-regulated among the pig and mouse species challenged with LPS compared to saline controls. To compare which differentially expressed genes are shared or unique between pig and mouse, the gene ids were first converted to human gene symbols following gprofiler2 gorth function (see Materials and methods).

### Table S7. Analysis of differentially expressed genes between tissues reveals largely consistent expression patterns of genes among tissues and a few tissue specific gene expression alterations.

Inference of differentially expressed genes between tissues to determine if genes are consistently up or down-regulated or show an opposite expression pattern between tissues.

### Table S8. Differentially expressed genes shared and unique among the brain, kidney, and blood.

Differentially expressed genes, adjusted p-value < 0.05, which are shared and unique among the brain, kidney, and blood following LPS challenge. Showing results for the samples were processed following the star/limma-voom pipeline.

### Table S9. Sample information.

Sample information including sample ID, group (control or LPS), weight, age, and various start and end clinical values.

### Table S10. Gene-level counts per million (CPM) data for each tissue.

Filtered to remove lowly expressed and keep only protein coding genes, gene-level counts per million (CPM) data for each tissue. This is the counts data used for the gene-level differential expression analysis with limma/voom pipeline.

### Table S11. Isoform-level transcripts per million (TPM) data for each tissue.

Filtered to remove lowly expressed and keep only protein coding transcripts, isoform-level transcripts per million (TPM) data for each tissue. This is the counts data used for the isoform-level differential expression analysis with Kallisto/Sleuth pipeline.

## ADDITIONAL FILE FIGURES

###

### Fig S1. Isoform-specific alterations in the brain reveal eight genes with opposite expression patterns.

**a)** Volcano plot showing isoform-level differential expression analysis in the brain of LPS pigs. **b)** Upset plot of DEG’s shared and unique between the isoform-level Kallisto/sleuth and gene-level star/limma pipelines (see Materials and methods) shows an overlap of gene sets between pipelines and unique DEG’s depending on the processing pipeline. **c)** from left to right are transcript structures, bootstrap estimated counts from kallisto, and log_2_ fold change (FC) for each isoform of *PSMC3*. There are five isoforms of *PSMC3*, of which one isoform is up-regulated (log_2_FC > 0 & adjusted p-value < 0.05) and another is (down-regulated log_2_FC < 0 & adjusted p-value < 0.05). The other three isoforms of *PSMC3* are not differentially expressed, with adjusted p-value > 0.05. **d)** *TSC2* gene has six isoforms expressed in the pig brain. One isoform is up-regulated, another is down-regulated, and four isoforms are not differentially expressed. **e)** *IL6*, and **f)** *CD248* have only one known isoform expressed in the pig brain data. *IL6* and *CD248* are up-regulated, adjusted p-value < 0.05, when the analysis is performed at the gene level via star/limma-voom pipeline, but are not called as differentially expressed adjusted p-value > 0.05 when the data is processed using the Kallisto/sleuth isoform-level pipeline.

### Fig S2. Multiple gene isoforms show opposite expression patterns in the kidney and are involved in neuron remodeling.

**a)** Volcano plot showing isoform-level differential expression within the kidney after LPS challenge. **b)** upset plot of DEG’s shared and unique between the isoform-level Kallisto/sleuth and gene-level star/limma pipelines within the kidney. Sixteen genes have multiple isoforms showing opposite expression patterns in the kidney. **c)** There are five isoforms of *ANKS1A* expressed in the kidney. From left to right are transcript structures, bootstrap estimated counts, and log_2_FC for each isoform. *ANKS1A* is thought to be involved in neuron remodeling and is called being down-regulated using the standard gene-level analysis. Still, here, via the isoform-level analysis, we observe isoforms of *ANKS1A* showing opposite expression patterns. **d)** *BRD4* has three isoforms expressed in the kidney, one up-regulated, another down-regulated, and another not differentially expressed. *BRD4* is up-regulated at the gene-level analysis. **e)** *TTC26* contains multiple isoforms with clear opposite expression patterns among the isoforms. *TTC26* is not differentially expressed at the gene-level analysis and may result from these opposite isoforms canceling each other out. **f)** *VMP* is called being up-regulated at the gene-level analysis; however, there are two up-regulated isoforms (log_2_FC > 1.6) and a down-regulated isoform (log_2_FC < 4) showing clear opposite expression patterns of *VMP* isoforms. **g)** *CEP350* gene contains three isoforms, with one isoform being called down-regulated in the kidney following LPS challenge; *CEP350* is also down-regulated at the gene-level analysis suggesting a predominant isoform of this gene.

### Fig S3. Genes involved in the signaling by Rho GTPases are up-regulated and down-regulated in the blood of LPS challenged pigs.

**a)** Volcano plot of DEG’s in whole blood for LPS (n = 4) versus control saline (n = 6).

**b)** down-regulated and up-regulated enriched functions in the blood reveal up-regulation of cell cycle, apoptosis, and signaling by Rho/Miro GTPases & RHOBTB3. Enrichment of down-regulated genes is involved in neutrophil degranulation, signaling by Rho GTPases, and hemostasis. **c) - g)** heatmaps showing gene expression, log_2_ counts per million (CPM), for the top up-regulated genes sorted by greatest to least log_2_FC within selected enrichment functions highlights the robust transcriptional alterations following LPS challenge. **h) - l)** heatmaps showing gene expression for the top fifteen down-regulated genes within selected enrichment functions.

### Fig S4. Numerous isoform alterations in the blood following LPS challenge.

**a)** Volcano plot of differentially expressed isoforms for LPS (n = 4) versus control saline (n = 6) from whole blood. **b)** Upset plot of differentially expressed genes shared and unique between the isoform-level Kallisto/sleuth and gene-level star/limma pipelines within the blood. **c)** from left to right are transcript structures, bootstrap estimated counts, and log_2_FC for each isoform of *ARHGEF10L*. Repeated for **d)** *CCR2*, **e)** *EVI5*, and **f)** *ICAM2* genes.

### Fig S5. Differentially expressed genes shared among the brain, kidney, and blood reveal consistent up-regulation of cytokine signaling, TNF signaling pathway, and PID IL12 2PATHWAY, and down-regulation of genes involved in the RHO GTPase cycle following LPS challenge.

**a)** Upset plot of DEG’s, adjusted p-value < 0.05, shows 11 down and 149 up-regulated genes that are common between the brain, kidney, and blood following LPS challenge. **b)** Enriched functions and predicted upstream regulators derived from differentially expressed genes reveal an up-regulation of cytokine signaling in the immune system and a down-regulation of the RHO GTPase cycle. **c)** heatmap showing log_2_ FC within each tissue for the three down-regulated genes within the RHO GTPase cycle enrichment function. **d - g)** heatmaps showing log_2_ FC within each tissue for the top up-regulated genes sorted by greatest to least fold change within selected up-regulated enrichment functions.
