# Supplementary material for: Commonly disrupted pathways in brain and kidney in a pig model of systemic endotoxemia
Source: J Neuroinflammation. 2024 Jan 4;21:9. doi: 10.1186/s12974-023-03002-6 (PMC10765757; doi:10.1186/s12974-023-03002-6)
Supplement: Supplementary file 2 — Additional file 2: Table S1. Clinical differences between control saline and LPS-treated pigs. Clinical data for each pig sample. Clinical data include weight, age, dose, the timing of the experiment, and vital signs [file 12974_2023_3002_MOESM2_ESM.pdf]

	CONTROL (SD)	LPS (SD)	difference between groups	
arterial lactate % change	3.67 (19.30)	227 (252.80)	t.test, p-val = 0.18	wilcox, p-val < 0.009
serum creatinine % change	25.5 (16.28)	131.5 (85.85)	t.test, p-val = 0.09	wilcox, p-val < 0.009
end urine output rate (mL/20 minutes)	19.42 (2.85)	3.87 (5.31)	t.test, p-val = 0.005	wilcox, p-val < 0.009
duration minutes	306 (3.74)	350.5 (163.55)	t.test, p-val = 0.62	wilcox, p-val = 1.00
mean age days	148.83 (15.33)	145.25 (7.41)	t.test, p-val = 0.64	wilcox, p-val = 0.76
mean weight kg	77.15 (2.01)	75.25 (3.23)	t.test, p-val = 0.35	wilcox, p-val = 0.45
start heart rate	86 (7.82)	92.5 (8.58)	t.test, p-val = 0.27	wilcox, p-val = 0.20
end heart rate	99.83 (18.83)	187 (26.29)	t.test, p-val = 0.002	wilcox, p-val = 0.01
start temperature	99.75 (1.11)	100.675 (1.52)	t.test, p-val = 0.34	wilcox, p-val = 0.52
end temperature	100.45 (1.52)	105.73 (4.35)	t.test, p-val = 0.002	wilcox, p-val = 0.038
start mean arterial pressure	93.22 (14.65)	105.09 (7.96)	t.test, p-val = 0.14	wilcox, p-val = 0.35
end mean arterial pressure	63.28 (4.17)	61.17 (8.18)	t.test, p-val = 0.66	wilcox, p-val = 0.76
